# Can Urbanization, Social and Spatial Disparities Help to Understand the Rise of Cardiometabolic Risk Factors in Bobo-Dioulasso? A Study in a Secondary City of Burkina Faso, West Africa

**DOI:** 10.3390/ijerph14040378

**Published:** 2017-04-04

**Authors:** Augustin Nawidimbasba Zeba, Marceline Téné Yaméogo, Somnoma Jean-Baptiste Tougouma, Daouda Kassié, Florence Fournet

**Affiliations:** 1 Institut de Recherche en Sciences de la Santé, Direction Régionale de l’Ouest, 01 BP 545 Bobo Dioulasso 01, Burkina Faso; florence.fournet@ird.fr; 2 Centre Hospitalier Universitaire Sorou Sanou, Université Polytechnique de Bobo-Dioulasso, 01 BP 676 Bobo-Dioulasso 01, Burkina Faso; teneline@gmail.com (M.T.Y.); tougjb@yahoo.fr (S.J.-B.T.); 3 LADYSS, Université Paris Ouest Nanterre la Défense, 92000 Nanterre, France; daoudakassie@hotmail.com; 4 UMR Mivegec (UM-CNRS 5290-IRD 224), Institut de Recherche pour le Développement, 34394 Montpellier, France

**Keywords:** chronic diseases, urbanization, health disparities, spatial sampling, medium-sized city, Bobo-Dioulasso

## Abstract

*Background*: Unplanned urbanization plays a key role in chronic disease growth. This population-based cross-sectional study assessed the occurrence of cardiometabolic risk factors in Bobo-Dioulasso and their association with urbanization conditions. *Methods*: Through spatial sampling, four Bobo-Dioulasso sub-spaces were selected for a population survey to measure the adult health status. Yéguéré, Dogona, Tounouma and Secteur 25 had very different urbanization conditions (position within the city; time of creation and healthcare structure access). The sample size was estimated at 1000 households (250 for each sub-space) in which one adult (35 to 59-year-old) was randomly selected. Finally, 860 adults were surveyed. Anthropometric, socioeconomic and clinical data were collected. Arterial blood pressure was measured and blood samples were collected to assess glycemia. *Results*: Weight, body mass index and waist circumference (mean values) and serum glycemia (83.4 mg/dL ± 4.62 mmol/L) were significantly higher in Tounouma, Dogona, and Secteur 25 than in Yéguéré; the poorest and most rural-like sub-space (*p* = 0.001). Overall, 43.2%, 40.5%, 5.3% and 60.9% of participants had overweight, hypertension, hyperglycemia and one or more cardiometabolic risk markers, respectively. *Conclusions*: Bobo-Dioulasso is unprepared to face this public health issue and urgent responses are needed to reduce the health risks associated with unplanned urbanization.

## 1. Introduction

The burden of chronic diseases is increasing worldwide, but particularly in developing countries [1,2] where young people [3] are concerned and lethality is higher. Several of these chronic diseases are directly related to nutrition transition, promoted by globalization and accelerated urbanization [4,5,6]. Indeed, people in developing countries are becoming more sedentary and progressively shifting towards a western-style diet with more animal products, refined grains, fat, salt and sugar and less fibers [7,8,9]. Consequently, chronic diseases are becoming one of the leading contributors to the disease, mortality and disability burden [3,10]. For instance, obesity prevalence in West Africa has increased by 114% from 1990 to 2005, and affects urban women 4.79 times more than men, as reported in a meta-analysis of data from this period [11].

Concomitantly, urbanization is also rapidly increasing. It is expected that more than 70% of people will be living in cities by 2050, and by 2030, 50% of the West African population should be located in urban cities [1]. Rapid urbanization is the main cause of the unplanned growth of cities. This is accompanied by a number of problems, such as environmental risks and health disparities, the consequences of which have not been determined yet. Urbanization and the rise of chronic diseases have been studied in cross-sectional studies in capital cities in the developing world. Conversely, little is known about urbanization and its health consequences in secondary cities. Bobo-Dioulasso, the second city of Burkina Faso, is a medium-sized city of West Africa with a rapidly progressing urbanization rate [12]. Hospital data have shown that the prevalence of obesity and hypertension is increasing in West Africa [13]; however, it is not known whether and how urban conditions contribute to this. The aim of the present study was to investigate the occurrence of cardiometabolic risk factors in different urban settings within Bobo-Dioulasso and to analyze their spatial distribution in relation with the socio-demographic characteristics.

## 2. Methods

### 2.1. Study Site

This study was carried out in Bobo-Dioulasso, a secondary city in the Western part of Burkina Faso. Bobo-Dioulasso has shown a relatively important and constant growth rate (4.7% per year, on average) since its discovery by colonialists. The population of Bobo-Dioulasso increased from 3000 to 50,000 inhabitants from the beginning of its colonization to its independence, and then from 230,000 inhabitants in 1975 to 310,000 in 1996 and 490,000 in 2006 [12]. This growth led to an important spatial expansion without specific building densification due to the absence of physical constraints. The city surface increased from 10.7 km^2^ in 1952 to 95.7 km^2^ in 2012.

This expansion was not without consequences on the urban planning, as exemplified by the irregular distribution of healthcare services in space and time. The installation of healthcare structures did not always follow the major phases of urban growth due to the different policies started by the government and international institutions. The number of healthcare structures rose from four before 1960 to 52 in 2012, without covering the entire urban space [14]. Similarly, differences concerning the access to drinking water have been observed (no data for non-regularized settlements and variable rates in regularized areas).

### 2.2. Spatial and Population Sampling

Several discriminating variables were retained to identify sub-spaces representative of the different urbanization types that could trigger health disparities in Bobo-Dioulasso population. Approaches based on Principal Component Analyses (PCA) and Hierarchical Ascendant Classification (HAC), previously validated in Dakar [15], were used to model the city typology. The chosen variables measured health vulnerability (building density, infrastructure level, flooding risk, and district age) and access to urban services (healthcare structures and drinking water). Five different urbanization classes were identified as reported by Kassié in his thesis [16]. Four sub-spaces were chosen among these different urbanization classes to maximize health disparities. They were selected based on their proximity to the center, period of urbanization and presence of infrastructures, such as schools and healthcare services that were identified during a street-by-street survey (Table 1).

After the identification of the sub-spaces to be surveyed, a simple spatial random sampling was used to select the plots from the cadastral map. This method ensured the comparability of data collected in the different districts.

A Pleiades satellite image of April 2012 with a very high resolution and the cadastral map of 2012 were used to identify and eliminate uninhabited parcels dedicated to administrative or commercial usage. Then, plots were randomly selected. For non-regularized areas, which are not recorded in the land registry map, plots were digitalized and their geographical coordinates used for the sampling. In each randomly selected plot, households and then the study participants were also randomly selected. The number of households to be surveyed and of participants was calculated in order to observe significant differences between sub-spaces at the 5% threshold with a precision of 2.6%, based on a diabetes prevalence of 5% (the smallest expected prevalence for diabetes in adults) (Table 1). A household was eligible if it included at least one eligible adult (35–59 years of age).

The geographical coordinates of the randomly selected plots were integrated in Garmin eTrex 10 handheld the Global Positioning System (GPS) units. Surveyors had to find these plots by using the procedure «Go to» of the GPS unit and with the help of maps in which these plots were shown. Each sub-space was subdivided in three parts and each part was assigned to a surveyor who had to cover it completely. This technique avoided selecting all the households in only one part of the study area. To be easily identified by the population, each surveyor had a badge and a work kit (GPS unit, map with the randomly selected points, forms for data collection, etc.).

In each selected plot, households were numbered and those that were eligible were identified. Then, the study households were selected randomly and their eligibility was verified. For each eligible household, the authorization of its head was obtained before data collection. Surveyors organized a second visit if the head was absent or if they had doubts about the age of the people to be surveyed, in order to give them time to gather accurate information. After three unsuccessful appointments, another household, already in the sampling list for that sub-space, was picked for the study. All collected and analyzed geographical data (aerial photographs, satellite images, geographical coordinates of urban infrastructures) were integrated in a geodatabase with a map projection WGS84 UTM 30 N.

The study sample size was estimated at 1000 households (250 for each sub-space). Finally, 3400 plots were randomly sampled among the 8812 plots identified from satellite images. A first random selection of 350 plots for each sub-space was carried out to offset the problems of uninhabited plots, wrong plot identification in the satellite images, absence of people, possible refusal and non-eligibility of households. Additional samplings were carried out after the removal of already visited plots. Finally, 860 adults were included based on the following inclusion criteria: age between 35 and 59 years and residence in the plot for at least six months. Pregnant women were excluded (Table 1).

### 2.3. Study Variables

After inclusion, each participant was interviewed to collect socio-demographic and lifestyle (diet and physical activity) data. At the end of each interview, anthropometric and clinical data and blood samples were also collected.

A Multiple Correspondence Analysis was performed to build a proxy of the income level using data on household assets, such as television, Digital Video Disc (DVD), fridge, motorbike, or car, house ownership, type of household toilet, electricity, type of cooking fuel, type of floor, roof and walls.

#### 2.3.1. Anthropometric Data

To obtain accurate body weight measurements, each participant was weighed in light clothing and without shoes using a portable electronic scale (Seca 803 Clara Scale; capacity 150 kg; graduation 100 g). Height was measured to the nearest 0.5 cm using a portable, locally built stadiometer with the subject standing upright on a flat surface without shoes and the back of the heels and the occiput against the stadiometer. Waist circumference (WC) was measured with a flexible steel tape (Gulick© measuring tape) to the nearest 0.1 cm at the midpoint between the lowest rib and the iliac crest with the participant standing and breathing normally [17]. Body weight, height and WC were measured twice and the mean of the two values was used for the analyses. Weight and height were used to calculate the body mass index (BMI) as follows: BMI = weight (kg) divided by height (m^2^). Based on the BMI values, participants were categorized as follows: BMI < 18.5: underweight; BMI 18.5–24.9: normal; BMI 25–29.9: overweight; BMI ≥ 30: obese [18]. Abdominal obesity was defined as a WC value ≥102 cm for men and ≥88 cm for women [19].

#### 2.3.2. Blood Pressure

Blood pressure was measured on the right arm by qualified medical doctors using an automatic blood pressure monitor OMRON M6-Comfort (OMRON HEALTHCARE Europe B.V., Hoofddorp, The Netherlands). Before the measurement, the participant has to rest in seated position for at least 10 min, and then blood pressure (systolic and diastolic) was measured twice with a 10 min interval between measures and the mean of the two values was retained. Participants with systolic blood pressure (SBP) ≥140 mmHg and/or diastolic blood pressure (DBP) ≥90 mmHg were considered as having hypertension [20,21]. People on hypertensive treatment were also included. Each participant with high blood pressure values was referred to a specialist for hypertension confirmation.

#### 2.3.3. Glycemia Testing

Glycemia was tested after 12-h fasting with the finger prick test. When fasting blood sugar was >110 mg/dL or >6 mmol/L [21], participants were considered at risk of hyperglycemia and were referred to a specialist for further confirmation by glycated hemoglobin measurement before being considered as having diabetes. People taking diabetes medications were also included.

#### 2.3.4. Cardiometabolic Risk Markers

Cardiometabolic risk factors considered in this study were: overweight or obesity, abdominal obesity, hypertension and hyperglycemia. Participants with at least one of these conditions were considered as having ”≥ 1 cardiometabolic risk marker”.

#### 2.3.5. Statistical Analyses

Data were analyzed with IBM-SPSS version 24.0 (IBM Corp, Armonk, NY, USA). Quantitative variables were expressed as mean ± standard deviation (SD), and categorical variables as percentages with 95% confidence intervals (CI). Differences between quantitative variables were assessed using the independent *t* test, and the Analysis of variance (ANOVA) test for comparison of more than two groups followed by the Bonferroni post hoc test. Differences between categorical variables were assessed with the χ^2^ test*.* The association between socio-demographic data, urbanization and cardiometabolic risk markers was assessed by multiple linear regression analysis. Differences were considered significant when *p* < 0.05.

#### 2.3.6. Ethical Considerations

The ethical committees of the Centre Muraz and of the Institut de Recherche en Sciences de la Santé examined the protocol and authorized this study (authorization no. A30-2013) that was carried out according to the Declaration of Helsinki guidelines.

In addition, the study aim and procedures were explained to each participant, each householder, the local authorities and the community at large. Prior to enrolment, each participant had to sign a written informed consent. The results of blood pressure and glycemia testing were given to each participant. Participants with abnormal results were referred to a specialist for diagnosis and treatment, the cost of which was supported by the research project.

## 3. Results

Overall, 860 adults were enrolled with a health survey response rate of 86%. The mean age of the enrolled adults was 43.4 ± 6.9 years (Table 2), and age was significantly higher among people living in Tounouma than in the other three sub-spaces (*p* = 0.02). Women were more numerous than men (56% vs. 44%) in the whole sample, and also within the sub-spaces, with the exception of Yéguéré where the proportion of men was significantly higher (*p* < 0.05). The percentage of participants with no education was significantly higher than that of people with formal education in the whole sample (*p* < 0.05) and also in Yéguéré compared with the other three sub-spaces (*p* = 0.001). Participants with high school or higher education level were more numerous in Tounouma and Secteur 25 than in the two other sub-spaces. For the whole sample, 33.2%, 33.6% and 33.2% of participants were in the low, medium and high income group, respectively. In Tounouma and Dogona, the distribution in the three income levels was comparable. Conversely, participants with high and middle income level were significantly more numerous in Secteur 25 than in Yéguéré, where participants with low income level were prevalent (*p* = 0.001).

### Cardiometabolic Risk Factors

In the whole sample, the mean weight was 68.9 ± 14.2 kg with a BMI of 24.6 ± 5.1 and a WC of 83.9 ± 11.8 cm. Women had significantly higher BMI (25.8 ± 5.4 vs. 23.2 ± 4.3; *p* = 0.0001) and WC (85.0 ± 12.4 vs. 82.7 ± 10.8; *p* = 0.004) values than men. The mean weight, BMI and WC values were significantly higher in Tounouma, Dogona, and Secteur 25 than in Yéguéré (*p* = 0.001). The mean glycemia was 83.4 ± 27.5 mg/dL in the whole sample, and was significantly higher in Tounouma, Dogona and Secteur 25 than in Yéguéré.

In the whole sample, 43.2%, 34.6%, 40.5% and 5.3% of participants had overweight/obesity, abdominal obesity, hypertension and hyperglycemia, respectively. The proportion of participants with overweight/obesity, abdominal obesity, hypertension and “≥1 cardiometabolic risk marker” was significantly higher in women than in men. The prevalence of hypertension and overweight/obesity was significantly lower in Yéguéré than in the other three sub-spaces. When all these cardiometabolic risk markers were clustered in the “≥1 cardiometabolic risk marker” category, 67.7% of participants fell in this group with no statistical difference among sub-spaces.

**Figure 1:**
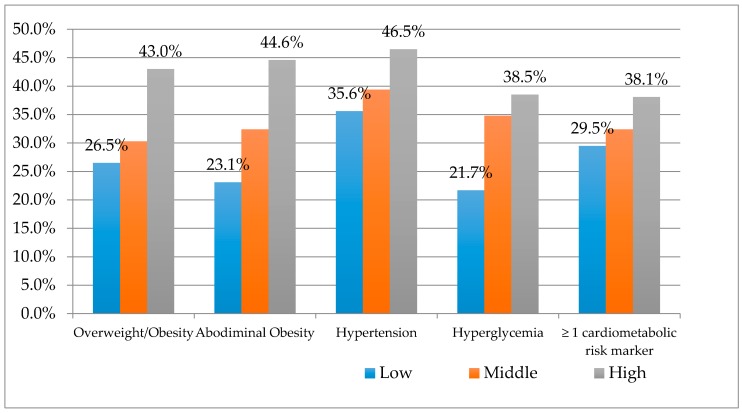
Cardiometabolic risk markers according to the socioeconomic status (low, middle and higher). In the figure change commas into points (ex. 5.0% into 5.0%); Abodiminal into Abdominal.

Overweight/obesity, abdominal obesity, hypertension, hyperglycemia and “≥1 cardiometabolic risk marker” (when clustering all the cardiometabolic risk factors) were significantly higher in the high income level group (Figure 1).

Analysis of the associations between cardiometabolic risk markers, socio-demographic characteristics and urban location using a multiple linear regression model showed that male sex was negatively and independently associated with BMI and WC (β = −0.249, *p* < 0.0001 and β = −0.089, *p* < 0.014, respectively) and positively and independently associated with SBP (β = 0.159, *p* = 0.001) (Table 3). Age was positively and independently associated with BMI, WC, hyperglycemia, SBP and DBP (β = 0.125, 0.134, 0.111, 0.352 and 0.241, respectively; *p* < 0.005). Living in Tounouma was positively and independently associated with all these five cardiometabolic risk markers (β = 0.136, 0.096, 0.108, 0.017 and 0.076, respectively; *p* < 0.005), while living in Yéguéré was negatively associated (β = −0.174, −0.106, −0.221, −0.016 and −0.061, respectively; *p* < 0.05). Low income was negatively associated with BM, WC, SBP and DBP, while high income was positively and independently associated with BMI, WC and DBP. High education level (secondary school and above) was positively associated with BMI, while low education level (primary school) was negatively associated with BMI.

## 4. Discussion

To our knowledge, this is one of the first studies in a medium-sized city of a developing country to explore the increase of cardiometabolic risk factors in relation with urbanization. The results show that in a developing country like Burkina Faso and even in secondary cities, such as Bobo-Dioulasso, chronic diseases are a health issue that is growing at an unanticipated pace. In 2003, a similar study carried out in Ouagadougou, the capital city of Burkina Faso, reported that 40.2% of participants had hypertension [22] and 33% overweight/obesity [23]. Like in the present study, high BMI (overweight/obesity) was more frequent in women, while high blood pressure in men [22,23,24,25,26]. These results indicate that the shift toward chronic diseases is taking place in Burkina Faso, particularly in its capital city, and highlight, as hypothesized, the role of urbanization in this process [27], for instance by promoting lifestyle changes, such as nutrition transition [9,28]. Although they are considered as diseases of affluent people in urban areas [29], in 2010 we found that cardiometabolic risk factors were frequent also among the poorest inhabitants of Ouagadougou [26]. This led to the hypothesis that due to the increasing urbanization, high prevalence of cardiometabolic risk factors could concern also Burkina Faso secondary cities, such as Bobo Dioulasso. The results of the present study confirm this hypothesis. Indeed, also in Bobo Dioulasso, the frequency of overweight/obesity, hypertension and hyperglycemia is increasing significantly and proportionally with the income level. This confirms the conclusion of the literature review by Monteiro et al. [30] that, in populations at early stages of the nutrition transition, excess weight occurs primarily among the more affluent, before progressing also to lower income groups. Moreover, the high frequency of cardiometabolic risk factors in the low income population, as observed in Yéguéré, indicates that not only the income level, but also urbanization contributes to the emergence of these health problems. The effect of urbanization on health can vary with the income level; however, it is debatable whether urbanization widens or narrows the health disparity between groups with different income level [31]. Urban lifestyle can put at risk low-income groups; however, urbanization could also flatten income level disparities and consequently, low income groups also may afford a lifestyle that promotes the appearance of chronic diseases [31,32], while higher income groups may face more risks associated with high stress levels, sedentary behavior and hypercaloric diets [33,34]. Our results indicate that both urbanization and socio-spatial disparities play a role in the increase of chronic diseases. Indeed, Yéguéré is the poorest of the four sub-spaces included in our survey, yet 37.2% and 29.1% of surveyed adults have hypertension and overweight/obesity, respectively. In Secteur 25, where we enrolled mostly people with high income level, 34.9% and 47.9% of participants have hypertension and overweight/obesity, respectively. This suggests that urbanization puts low-income level populations in contact with factors that can increase the cardiometabolic risk burden. It also suggests that urbanization in Bobo Dioulasso has not yet reached a level that allows the high income group to be sheltered from these same factors.

Similar studies reported a positive association between the income level and cardiometabolic risk markers, and demonstrated that this was the results of diet changes because high income level populations can afford more expensive food, such meat/poultry, dairy products and sweets/sweet drinks that are known to be correlated with cardiometabolic risk markers [35,36].

Moreover, Ntandou et al. [37] reported that higher physical activity levels in rural areas protect against cardiometabolic risk factors, while the more sedentary lifestyle in urban areas explains their appearance in urban populations. This could help understanding why living in Yéguéré, the poorest and most rural-like sub-space in our study, was independently and negatively associated with BMI, glycemia, and blood pressure, while living in Tounouma, a richer and more urbanized sub-space, was positively and independently associated with the same cardiometabolic risk factors.

The study is not exempt of limitations. One of the obvious limitations is the study cross-sectional design that does not allow drawing any inference on the causal relationships between variables. Moreover, sampling and sample size were not computed to be representative of Bobo-Dioulasso inhabitants and for this reason, our results cannot be extrapolated to the whole urban population of Burkina Faso without caution. Another limitation is the absence of data on blood lipids, which could have given a better description of the study population and also helped identifying people with metabolic syndrome. However, despite these limitations, this study provides useful data that can help explaining the increase of cardiometabolic risk markers in urban populations and elicit more investigations to better understand the link with urbanization.

## 5. Conclusions

This study shows that the increase of diet-related cardiometabolic risk markers in adults is a public health concern even in a medium-sized city, such as Bobo-Dioulasso. Urbanization certainly plays an increasing and important role due to the improved sanitation and healthier environment, higher socioeconomic status and better access to health services for both low and high income groups. Unfortunately, unplanned urbanization is associated with negative effects, while the positive contribution of urbanization to health and wellbeing is mitigated. This study shows how the simple description of settlement and urbanization conditions could help understanding health conditions in Bobo-Dioulasso. Although our results do not fully explain the reasons underlying the increase of cardiometabolic risk markers in this secondary city, they suggest that unplanned urbanization play an important role. This issue could be directly addressed by a dedicated urbanization policy and through well-designed health education interventions.

## Figures and Tables

**Table 1:** Features of the chosen sub-spaces and distribution of surveyed adults in the different sub-spaces.

	Proximity to the Center	Period of Urbanization	Presence of Infrastructures	Surveyed Adults		
				Men	Women	Total
Tounouma	+++	Old	++	111	142	253
Dogona	++	Old	+++	84	109	193
Secteur 25	−	Recent	+	79	136	215
Yéguéré	+	Recent	−	104	95	199
Total				378	482	860

The more + the more, the sub-space is close the center of the city with more infrastructures; the – sign shows that the sub-space if far from the city center and has no infrastructures.

**Table 2:** Socio-demographic characteristics, nutritional status and cardiometabolic risk markers by sector of residence.

	All (*n* = 816)		Tounouma (*n* = 253)		Dogona (*n* = 193)		Secteur 25 (*n* = 215)		Yéguéré (*n* = 199)		*p*
	Mean or %	SD or 95% CI	Mean or %	SD or 95% CI	Mean or %	SD or 95% CI	Mean or %	SD or 95% CI	Mean or %	SD or 95% CI	
Age (years)	43.2	6.9	44.2	7.3	43.5	6.9	42.1	6.6	43.0	6.0	0.02
Sex											
Women	56.0	(52.7; 59.3)	56.1	(50.0; 62.2)	56.5	(49.5; 63.5)	63.3	(56.9; 69.7)	47.7	(40.8; 54.6)	0.076
Men	44.0	(40.7; 47.3)	43.9	(37.8; 50.0)	43.5	36.5; 50.5)	36.7	(30.3; 43.1)	52.3	(45.4; 59.2)	0.076
Formal education											
None	44.8	(39.9; 49.7)	35.6	(29.7; 41.5)	43.5	(36.5; 50.5)	49.8	(43.1; 56.5)	52.3	(45.4; 59.2)	0.001
Men	24.3	(20.0; 28.6)	18.0	(10.0; 26.0)	18.3	(9.9; 26.7)	22.6	(14.6; 30.6)	36.2	(27.1; 45.3)	0.426
Women	75.7	(71.4; 80.0)	82.0	(74.0; 90.0)	81.7	(73.3; 90.3)	77.4	(69.4; 85.4)	63.8	(54.7; 72.9)	0.426
Primary school	38.5	(33.3; 43.7)	37.9	(32.0; 43.8)	44.0	(37.0; 51.0)	32.1	(25.9; 38.3)	40.7	(33.9; 47.5)	0.001
Men	58.8	(53.5; 64.1)	56.3	(46.4; 66.2)	60.0	(49.6; 70.4)	46.4	(34.6; 58.2)	71.3	(61.4; 81.2)	0.1
Women	41.2	(35.9; 46.5)	43.7	33.8; 53.6)	40.0	(29.6; 50.4)	53.6	(41.8; 55.4)	28.7	(18.8; 38.6)	0.1
Secondary school & above	16.7	(4.6; 22.8)	26.5	(21.1; 31.9)	12.4	(7.8; 17.0)	18.1	(13.0; 23.1)	7.0	(3.5; 10.5)	0.001
Men	61.1	(53.2; 69.0)	59.7	(48.0; 71.4)	66.7	(47.9; 85.5)	59.0	(43.6; 74.4)	64.3	(39.3; 92.3)	0.918
Women	38.9	(31.4; 46.8)	40.3	(28.6; 52.0)	33.3	(14.5; 52.6)	41.0	(25.6; 56.4)	35.7	(10.7; 60.7)	0.918
Income level											
Low	33.2	(27.8; 38.6)	29.2	(23.6; 34.8)	38.0	(31.1; 44.9)	5.6	(2.5; 8.7)	63.3	(56.6; 70.0)	0.001
Men	46.1	(40.3; 51.9)	42.5	(31.2; 53.8)	43.8	(32.4; 55.2)	25.0	(0.5; 49.5)	51.6	(42.9; 60.3)	0.239
Women	53.9	(48.1; 59.7)	57.5	(46.2; 68.8)	56.2	(44.8; 67.6)	75.0	(50.5; 99.5)	48.4	(39.7; 57.1)	0.239
Middle	33.6	(28.2; 39.0)	36.0	(30.1; 41.9)	43.2	(36.2; 50.2)	33.3	(27.0; 39.9)	21.1	(15.4; 26.8)	0.001
Men	42.2	(36.5; 47.9)	45.6	(35.3; 55.9)	42.2	(31.6; 52.8)	31.9	(21.2; 42.6)	52.4	(37.4; 67.4)	0.151
Women	57.8	(52.1; 63.5)	54.4	(44.1; 64.7)	57.8	(47.2; 68.4)	68.1	(57.4; 78.8)	(47.6	(32.6; 47.6)	0.151
High	33.2	(27.8; 38.6)	34.8	(28.9; 40.7)	18.8	(13.3; 24.3)	60.7	(54.2; 67.2)	15.6	(10.6; 20.6)	0.001
Men	44.0	(38.3; 49.7)	43.7	(33.3; 54.0)	47.2	(30.9; 63.5)	40.8	(32.4; 49.2)	54.8	(37.8; 72.3)	0.535
Women	56	(50.3; 61.7)	56.3	(45.9; 66.7)	52.8	(36.5; 69.1)	59.2	(50.8; 67.6)	45.2	(27.7; 62.7)	0.535
Height (cm)	167.5	8.9	167.5	9.1	166.9	9.6	166.9	8.2	168.4	8.5	0.284
Men	173.5	8.2	174.0	8.1	172.1	11.1	174.3	6.3	173.5	6.9	0.0001
Women	162.7	6.1	162.4	6.3	163.1	5.9	162.7	5.9	162.8	6.3	0.0001
Weight (kg)	68.9	14.2	72.0	16.7	67.8	12.3	69.6	13.6	65.3	11.9	0.001
Men	69.7	12.8	72.1	15.4	68.4	12.4	71.0	11.5	67.3	10.7	0.173
Women	68.3	15.1	72.0	17.7	67.3	12.4	68.8	14.7	63.2	12.9	0.173
BMI kg/m^2^	24.6	5.1	25.7	5.9	24.5	5.1	25.0	4.8	23.0	3.9	0.001
Men	23.2	4.3	23.8	4.8	23.4	5.3	23.4	3.7	22.3	3.2	0.0001
Women	25.8	5.4	27.2	6.2	25.3	4.7	26.0	5.2	23.8	4.5	0.0001
WC (cm)	83.9	11.8	86.1	13.2	82.8	11.9	84.7	11.3	81.5	9.6	0.001
Men	82.7	10.8	84.1	12.1	80.9	10.9	84.1	10.9	81.5	8.9	0.004
Women	85.0	12.4	87.7	13.7	84.2	12.5	85.2	11.6	81.4	10.5	0.004
DBP	83.7	13.7	85.3	14.6	83.7	13.3	83.3	13.6	82.2	12.7	0.095
Men	83.0	13.7	86.0	16.0	81.7	12.8	81.3	11.8	82.2	12.7	0.181
Women	84.3	13.6	84.7	13.7	85.3	13.5	84.4	14.4	82.2	12.7	0.181
SBP	126.2	19.7	129.2	23.7	124.6	17.7	124.3	21.6	135.8	19.7	0.083
Men	128.1	20.0	132.5	24.2	125.1	16.3	123.7	17.8	129.0	18.1	0.021
Women	124.7	22.0	126.6	23.2	124.3	18.7	124.7	23.6	122.3	20.8	0.021
Blood sugar (mg/dL)	83.4	27.5	87.9	33.2	88.9	22.3	83.4	27.0	72.3	20.7	0.0001
Men	84.0	23.6	86.2	17.3	92.7	16.7	89.1	35.3	70.9	17.1	0.682
Women	83.0	30.3	89.2	41.6	86.1	25.5	80.0	20.2	73.9	24.1	0.682
Hypertension	40.5	(37.2; 43.7)	48.2	(42.0; 54.3)	39.9	(32.9; 46.8)	34.9	(28.4; 41.3)	37.2	(30.5; 43.9)	0.003
Men	42.2	(37.0; 47.4)	46.7	(37.9; 55.5)	37.7	(27.0; 48.4)	32.0	(21.5; 42.5)	50.0	(38.6; 61.4)	0.223
Women	57.8	(52.6; 63.0)	53.3	(44.5; 62.1)	62.3	(51.9; 73.0)	68.0	(57.5; 78.5)	50.0	(38.6; 61.4)	0.223
Overweight/Obesity	43.2	(39.9; 46.5)	50.6	(44.4; 56.7)	42.9	(35.9; 49.8)	47.9	(41.1; 54.6)	29.1	(22.7; 35.4)	0.001
Men	32.2	(27.5; 36.9)	30.5	(22.6; 38.4)	34.6	(24.3; 44.9)	28.2	(19.5; 36.9)	39.7	(27.2; 52.2)	<0.001
Women	67.8	(63.1; 72.5)	69.5	(61.6; 77.4)	65.4	(55.1; 75.7)	71.8	(63.1; 80.5)	60.3	(47.8; 72.8)	<0.001
Abdominal obesity	34.6	(31.4; 37.8)	40.3	(34.3; 46.3)	30.9	(24.3; 37.5)	38.6	(32.1;45.1)	26.6	(20.5; 32.7)	0.008
Men	37.5	(32.0; 43.0)	34.3	(25.1; 43.5)	43.5	(22.3; 46.7)	33.7	(23.5; 43.9)	52.8	(39.4; 66.2)	0.004
Women	62.5	(57.0; 68.0)	65.7	(56.5; 74.9)	65.5	(53.3; 77.7)	66.3	(56.1; 76.5)	47.2	(33.8; 60.6)	0.004
Hyperglycemia	5.3	(3.8; 6.7)	6.7	(3.6; 9.7)	6.7	(3.1; 10.2)	6.0	(2.8; 9.2)	1.5	(0.2; 3.2)	0.007
Men	50.0	(35.6; 64.4)	52.9	(29.2; 76.6)	53.8	(26.7; 80.9)	46.2	(19.1; 73.3)	33.3	(10.0; 56.6)	0.242
Women	50.0	(35.6; 64.4)	47.1	(23.4; 70.8)	46.2	(19.1; 73.3)	53.8	(26.7; 80.9)	66.7	(43.4; 90.0)	0.242
≥1 cardiometabolic risk marker	62.7	(59.5; 65.9)	69.2	(63.5; 74.8)	60.6	(53.7; 67.4)	60.5	(53.9; 67.1)	51.3	(44.3; 58.2)	0.269
Men	39.7	(35.6; 43.8)	39.2	(32.0; 46.4)	39.2	(30.5; 47.9)	34.1	(26.1; 42.1)	48.1	(38.7; 57.5)	0.001
Women	60.3	(56.2; 64.4)	60.8	(53.6; 68.0)	60.8	(52.1; 69.5)	65.9	(57.9; 73.9)	51.9	(42.5; 61.3)	0.001

BMI: Body mass index; WC: waist circumference; SBP: systolic blood pressure; DBP: diastolic blood pressure.

**Table 3:** Multiple linear regression analysis of the association between cardiometabolic risk factors and location and socio-demographic data.

	Cardiometabolic Risk Factors									
	BMI	*p* Value	WC	*p* Value	Hyperglycemia	*p* Value	SBP	*p* Value	DBP	*p* Value
Adjusted R^2^	0.115	<0.0001	0.64	<0.0001	0.059	<0.0001	0.146	<0.0001	0.063	<0.0001
Sex (♀ = 0; ♂ = 1)	−0.249	<0.0001	−0.089	0.014	0.016	0.318	0.159	0.001	−0.001	0.985
Age	0.125	<0.0001	0.134	<0.0001	0.111	0.001	0.352	<0.0001	0.241	<0.0001
Sub-space										
Dogona	−0.015	0.332	−0.87	0.028	0.104	0.001	−0.075	0.053	−0.036	0.415
Secteur 25	0.042	0.112	−0.075	0.07	0.003	0.468	−0.081	0.048	−0.012	0.363
Tounouma	0.136	<0.0001	0.096	0.028	0.108	0.001	0.017	0.007	0.076	0.025
Yéguéré	−0.174	<0.0001	−0.106	0.011	−0.221	<0.0001	−0.016	0.005	−0.061	0.038
Income group										
Low	−0.138	<0.0001	−0.168	<0.0001	−0.037	0.140	−0.061	0.039	−0.077	0.012
Middle	−0.029	0.199	−0.127	0.001	0.006	0.428	0.038	0.345	0.001	0.485
High	0.167	<0.0001	0.169	<0.0001	0.031	0.185	0.057	0.186	0.076	0.014
Education level										
No formal education	0.012	0.358	−0.024	0.538	−0.023	0.255	−0.021	0.268	−0.009	0.397
Primary school	−0.061	0.038	0.023	0.538	−0.024	0.240	−0.028	0.459	−0.027	0.213
Secondary school & above	0.063	0.034	0.030	0.423	0.062	0.036	0.014	0.721	0.048	0.083

BMI: Body mass index; WC: waist circumference; SBP: systolic blood pressure; DBP: diastolic blood pressure. For blood pressure, 58 participants with known hypertension were excluded. For glycemia, 7 participants with known diabetes were excluded.
